# Efficient base editing with high precision in rabbits using YFE-BE4max

**DOI:** 10.1038/s41419-020-2244-3

**Published:** 2020-01-20

**Authors:** Zhiquan Liu, Siyu Chen, Huanhuan Shan, Yingqi Jia, Mao Chen, Yuning Song, Liangxue Lai, Zhanjun Li

**Affiliations:** 1 Key Laboratory of Zoonosis Research, Ministry of Education, College of Animal Science, Jilin University, Changchun, 130062 China; 2 CAS Key Laboratory of Regenerative Biology, Guangdong Provincial Key Laboratory of Stem Cell and Regenerative Medicine, South China Institute for Stem Cell Biology and Regenerative Medicine, Guangzhou Institutes of Biomedicine and Health, Chinese Academy of Sciences, Guangzhou, 510530 China; 3 Guangzhou Regenerative Medicine and Health Guang Dong Laboratory (GRMH-GDL), Guangzhou, 510005 China; 4 Institute for Stem Cell and Regeneration, Chinese Academy of Sciences, Beijing, 100101 China

**Keywords:** Gene delivery, DNA damage and repair

## Abstract

Cytidine base editors, composed of a cytidine deaminase fused to Cas9 nickase, enable efficient C-to-T conversion in various organisms. However, current base editors suffer from severe trade-off between editing efficiency and precision. Here, based on rationally mutated cytidine deaminase domain, we develop a new base editor, YFE-BE4max, effectively narrow the editing width to as little as approximately three nucleotides while maintaining high efficiency in rabbits. Moreover, YFE-BE4max successfully mediated the *Tyr* p. Q68Stop and *Lmna* p. G607G mutation in F0 rabbit with high efficiency and precision, which precisely recapitulates the pathological features of human OCA1 and HGPS, respectively. Collectively, YFE-BE4max system provide promising tools to perform efficient base editing with high precision in rabbits and enhances its capacity to precisely model human diseases.

## Introduction

The clustered regularly interspaced short palindromic repeat (CRISPR) system has exhibited powerful genome manipulation capability in various organisms^1,2^. Base editing is a revolutionary technology based on the CRISPR platform, which can achieve targeted C-to-T conversion without generating DNA double-strand breaks or requiring a donor template^3^. The most common base editing system, base editor 3 (BE3), consists of rat APOBEC1 (rA1) fused with a *Streptococcus pyogenes* Cas9 (SpCas9) nickase and uracil glycosylase inhibitor^3^. Efficient editing by BE3 requires the presence of a protospacer-adjacent motif (PAM) of NGG that places the target C within an ~5-nucleotide window near the PAM-distal end of the protospacer (positions 4–8, counting the PAM as positions 21–23) in human cells^3,4^.

BE3 can potentially induce unwanted C-to-T substitutions when more than one C is present in the large ~5-nucleotide window, which can negatively affect the precision of targeted base editing. Therefore, such system is not ideal for precise disease modeling and gene therapy when accurate single C substitution is required. To overcome this limitation, Liu et al optimized rA1 with mutant deaminase domains (YE base editors), representatively termed YE1 (W90Y + R126E) and YEE (W90Y + R126E + R132E), to effectively narrow the width of the editing window from ~5 nucleotides to as little as 1–2 nucleotides in human cells^4^. YEE-BE3 showed better accuracy than YE1-BE3, but has lowered editing efficiency at target loci^4,5^.

In this report, by rationally engineering Cas9-cytidine deaminase fusions, we optimized cytidine base editors with high precision. In addition, these new base editors were successfully applied to produce Founder (F0) rabbits, demonstrating their high efficiency and precision in inducing C-to-T conversions at multiple endogenous loci in organisms.

## Results

### Comparison of C-to-T base editing using BE4-Gam and other mutated deaminase-Cas9 fusions in rabbit embryos

**Fig. 1:**
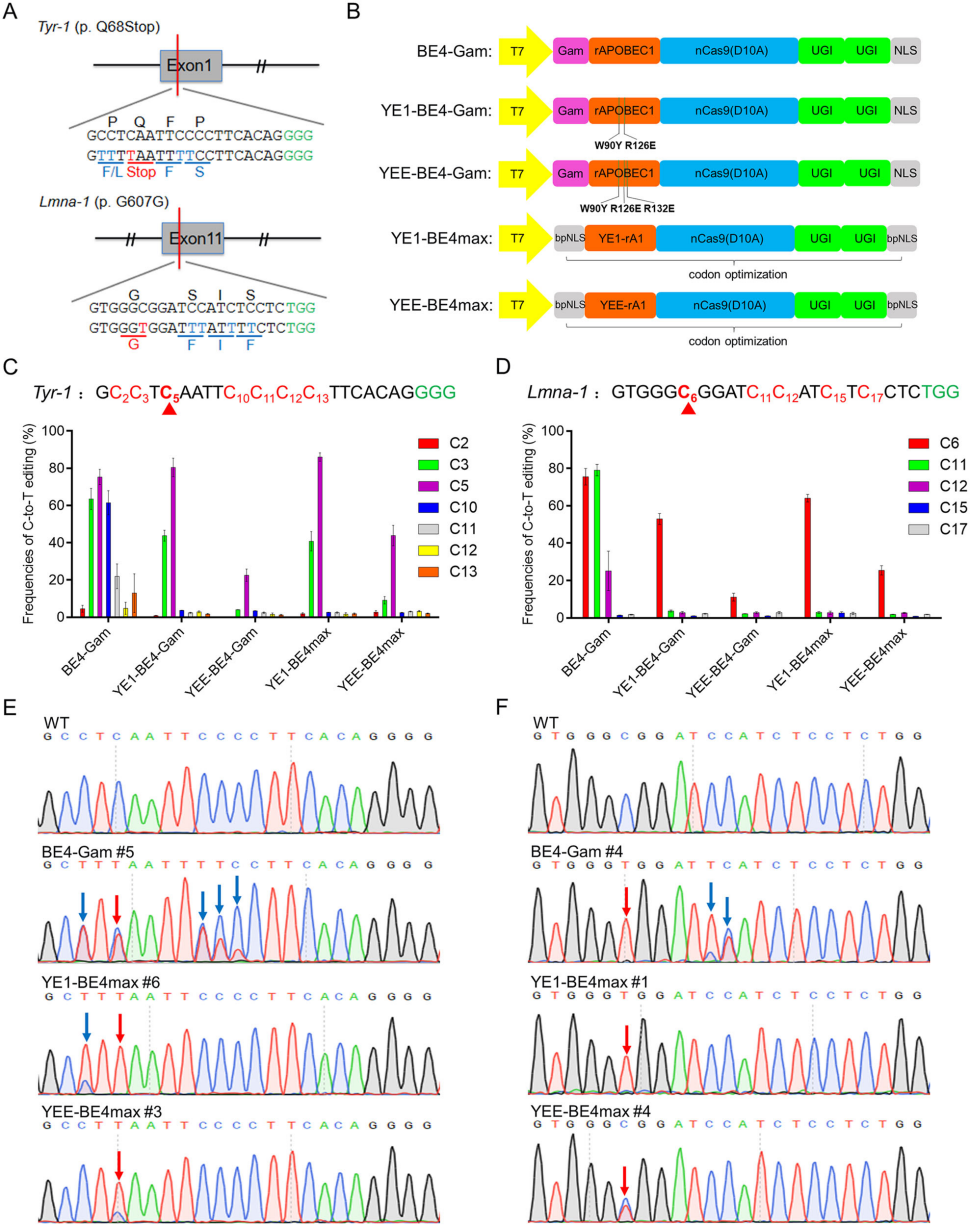
Comparison of C-to-T base editing using BE4-Gam and other mutated deaminase-Cas9 fusions in rabbit embryos. **a** The target sequence at the *Tyr-1* and *Lmna-1* sites. Target sequence (black), PAM region (green). The target C-to-T-editing sites or potential bystander mutations are marked in red or blue, respectively. The relevant codon identities at the target site are presented under the DNA sequence. **b** Schematic representation of the five base editors’ architecture. The point mutations that mutated in rAPOBEC1 are indicated by the green lines. YE1 (W90Y+R126E), YEE (W90Y+R126E+R132E). **c**, **d** Frequencies of targeted single C-to-T conversion at *Tyr-1* **c** and *Lmna-1* **d** by five base editors in rabbit embryos. The red triangle indicates the target C of the desired mutation. Target sequence (black), PAM region (green), mutated Cs (red, counting the PAM as positions 21–23). **e**, **f** Representative sequencing chromatograms at *Tyr-1* **e** and *Lmna-1* **f** of edited rabbit blastocyst using three base editors. Targeted base editing (red arrows), bystander base editing (blue arrows). *WT*, wild type.

Our previous study demonstrated that BE3 system can be used to mimic human pathologies by efficiently mediate C-to-T conversions in rabbits^6^. However, it frequently induce high proportions of bystander mutations at target loci, especially at *Tyr-1* p. Q68Stop and *Lmna-1* p. G607G^6^. The results of deep sequencing analysis indicated that unwanted C-to-T mutations were observed in a large width from C2 to C11 or from C6 to C17 at *Tyr-1* or *Lmna-1*, respectively (Fig. S1). In particular, a high proportion of bystander C-to-T mutations lead to changes in amino acids such as bystander C3 (50.83%, p. P67L) at *Tyr-1* and bystander C11 (77.00%, p. S609F) at *Lmna-1*, thus may potentially confound the correspondence between the genotype and phenotype of mutants (Figs. 1a and S1). The bystander mutations may arise from the relatively wide editing window of BE3, and the high activity of APOBEC1 likely contributes to the deamination of multiple Cs per DNA-binding event, consistent with previous reports in human cells^3^.

To reduce the bystander mutations, we first tested the current representative YE1 and YEE systems in rabbit embryos at *Tyr-1* and *Lmna-1* as a proof of concept. The YE1-BE4-Gam and YEE-BE4-Gam vectors were constructed by introducing the corresponding point mutations into BE4-Gam, a new version of base editor that has been demonstrated to have identical efficiency to traditional BE3 and higher product purity^6,7^ (Fig. 1b). Base editing frequencies were evaluated from Sanger sequence chromatograms of each blastocyst using EditR, a robust and inexpensive base editing quantification software has been widely used in previous reports^6,8,9^. Notably, YE1-BE4-Gam maintained activity comparable to that of BE4-Gam at the target C5 (80.47 ± 13.25% versus 75.28 ± 9.38%), whereas minimized deamination activity at other positions, thus narrowing the editing width from C3 to C5 at *Tyr-1* (Fig. 1c). In addition, at *Lmna-1*, YE1-BE4-Gam almost eliminated the bystander activity at C11 and C12 but did modestly reduce the editing frequency at target C6 (52.95 ± 7.14% versus 75.55 ± 10.86%; *p* = 0.11) (Fig. 1d). However, YEE-BE4-Gam showed significantly reduced editing efficiency at both target loci (22.52 ± 8.21%; *p* < 0.01 at *Tyr-1* and 11.10 ± 5.07%; *p* < 0.001 at *Lmna-1*) although nearly free from bystander mutations (Fig. 1c, d). This outcome is consistent with the low efficiency of YEE variant that previously reported in cultured cells^4,5,10^.

A recently developed base editor, BE4max, can significantly increase editing efficiency by modifying nuclear localization signals and codon usage^11^. Subsequently, we exploited this method to optimize the efficiency of YE base editors, and constructed YE1-BE4max and YEE-BE4max systems (Fig. 1b). In comparison with YE1-BE4-Gam, YE1-BE4max showed higher activity at *Tyr-1* (86.05 ± 5.46% versus 80.47 ± 13.25%) and *Lmna-1* (64.07 ± 5.02% versus 52.95 ± 7.14%) (Fig. 1c, d). The YEE-BE4max showed surpassing activity at both loci compared with that of YEE-BE4-Gam, but its editing efficiency is relatively inferior (43.88 ± 13.44% at *Tyr-1* and 25.46 ± 5.52% at *Lmna-1*) compared with that of conventional BE4-Gam (Fig. 1c, d). In addition, all five base editors exhibited relatively low frequencies of indels and non-C-to-T conversions, consistent with the high product purity of the BE4 system in previous reports^6,7^ (Fig. S2). Overall, these results indicated that YE1 base editors (YE1-BE4-Gam and YE1-BE4max) narrowed the editing window to ~C3–C6 with comparable efficiency, and YEE base editors (YEE-BE4-Gam and YEE-BE4max) narrowed the editing window to ~C5–C6 with notably decreased activity at the two loci (Fig. 1e, f).

### Optimization of deaminase domain improves efficiency and precision

**Fig. 2:**
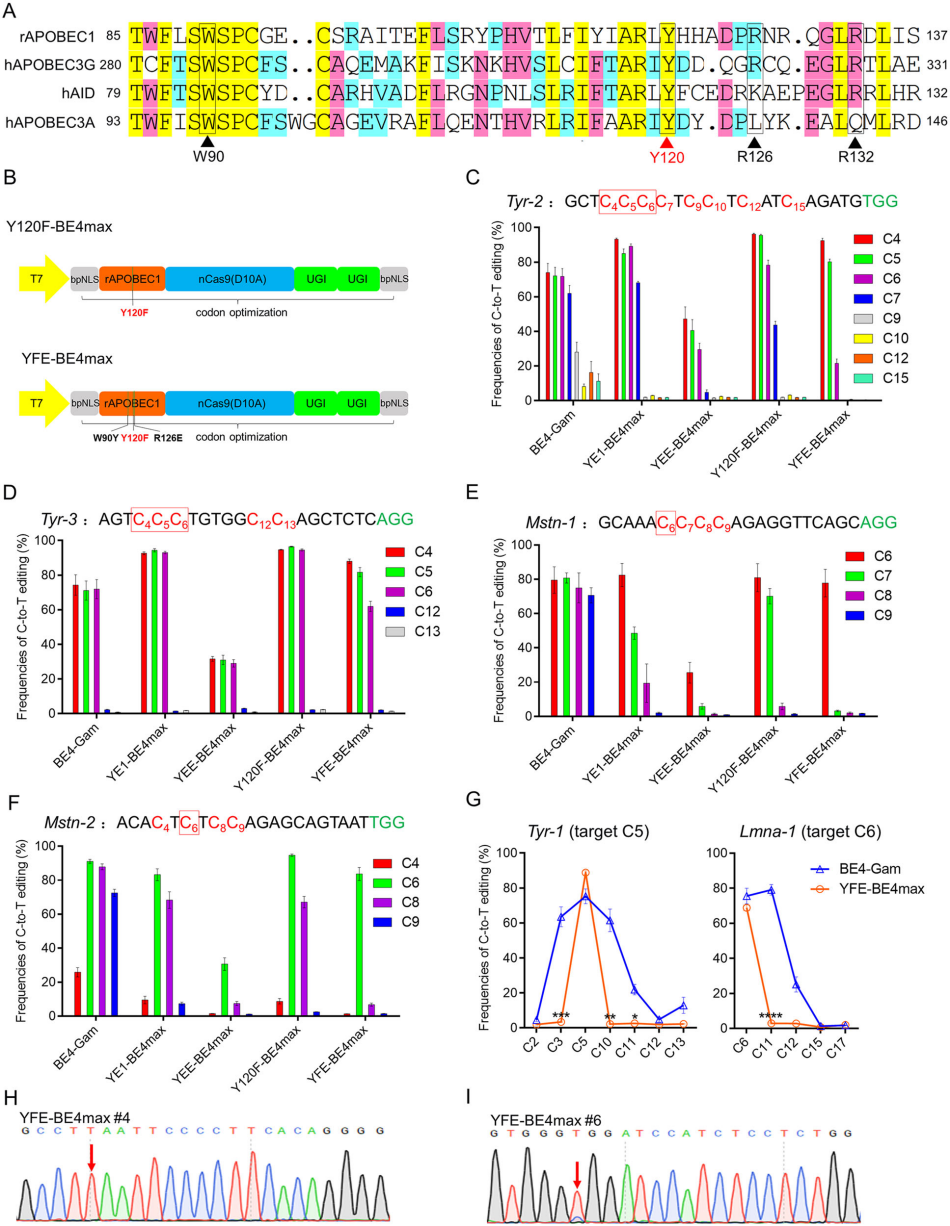
Base editors with rationally engineered cytidine deaminase domains exhibit narrowed editing windows with maintained efficiency in rabbit embryos. **a** Amino-acid alignments of rAPOBEC1 with three human APOBEC/AID family members. W90, R126, and R132 or Y120 are shown in black or red, respectively. **b** Schematic representation of Y120F-BE4max and YFE-BE4max. **c**–**f** Frequencies of single C-to-T conversion at *Tyr-2* **c**, *Tyr-3* **d**, *Mstn-1* **e**, and *Mstn-2* **f** by five base editors in rabbit embryos. Target sequence (black), PAM region (green), mutated Cs (red, counting PAM as positions 21–23). The red rectangle indicates the core window of YFE-BE4max. **g** Comparison of frequencies of single C-to-T conversions using BE4-Gam and YFE-BE4max at *Tyr-1* and *Lmna-1* in rabbit embryos. **h**, **i** Representative sequencing chromatograms at *Tyr-1* **h** and *Lmna-1* **i** of edited rabbit blastocyst using YFE-BE4max. Targeted base editing (red arrows).

The point mutations in rA1 of YE1 and YEE were designed to slow its kinetic rate, thereby reduced the catalytic efficiency of the deaminase domain^4^. In fact, W90Y and R126E mutations in YE1 can specifically narrow the size of editing window while maintain efficiency, but an additional R132E mutation in YEE can dramatically reduce the editing efficiency. Thus, rationally engineering cytidine deaminases by replacing the deleterious R132E mutation may be an effective strategy to improve the base editors’ efficiency and precision simultaneously. Moreover, the mutation of residue Y130 partially reduced the deamination activity of human APOBEC3A (hA3A), thereby narrowing the editing width in a recent study^12^. Y130 in hA3A, equivalent to Y120 in rA1, is near the active center and crucial for contacting the target cytosine^13,14^. Furthermore, Y120 in rA1 is a highly conserved site among multiple cytidine deaminases, as shown by alignment of the amino-acid sequences (Fig. 2a).

Based on these findings, we introduced similar Y120F mutations into rA1 (Y120F-BE4max) (Fig. 2b). Four target sites with a subset of Cs present in the activity window were selected to verify the hypothesis. The control group, BE4-Gam, showed a large editing window mainly from C4 to C9 (~6 nt) and even induced the widest range of mutations spanning from C4 to C15 at *Tyr-2* (Fig. 2c–f). In contrast, YE1-BE4max exhibited ideal efficiency comparable to that of BE4-Gam, but narrowed the editing width to ~4 nt (C4-C7) at *Tyr-2* and ~3 nt at the other three sites (Fig. 2c–f). YEE-BE4max narrowed the editing window to ~3-nt (C4-C6) at *Tyr-2* and *Tyr-3*, even to ~1 nt (C6) at *Mstn-1* and *Mstn-2*, but its editing efficiency is significantly reduced. Notably, modified Y120F-BE4max maintained high activity and decreased bystander mutations with narrowed editing window (~3–4 nt) at target sites (Fig. 2c–f), which indicates that Y120F mutation has a similar effect of narrowing editing window as the combined effect of W90Y and R126E in YE1, and does not affect editing efficiency. Encouraged by this result, we further combined the Y120F and YE1 mutations to produce a triply mutated base editor, termed YFE-BE4max (W90Y + Y120F + R126E) (Fig. 2b). Remarkably, YFE-BE4max showed similar maximal editing efficiency as Y120F-BE4max, but substantially narrowed editing window width to ~3 nt on both *Tyr-2* and *Tyr-3* (Fig. 2c, d). In particular, induced C-to-T conversion at C6 almost accurately, with few bystander mutations at both *Mstn-1* and *Mstn-2* (Fig. 2e, f). Moreover, YFE-BE4max efficiently induced precise base editing at target Cs at both *Tyr-1* and *Lmna-1* with minimized bystander activities in surrounding regions (Fig. 2g–i). Furthermore, by conducting detailed analysis, the YFE-BE4max can efficiently induce base editing in a narrower window (~3 nt, positions 4–6 in the sgRNA target site) compared with that of initial BE4-Gam (~9 nt, positions 3–11) (Fig. S3). Taken together, these results demonstrated that YFE-BE4max can induce efficient base editing within a narrow window (~3 nt) in rabbit embryos, thus it can serve as a powerful tool to mediate targeted point mutations.

### Precise base conversion at *Tyr* to recapitulate human albinism

**Fig. 3:**
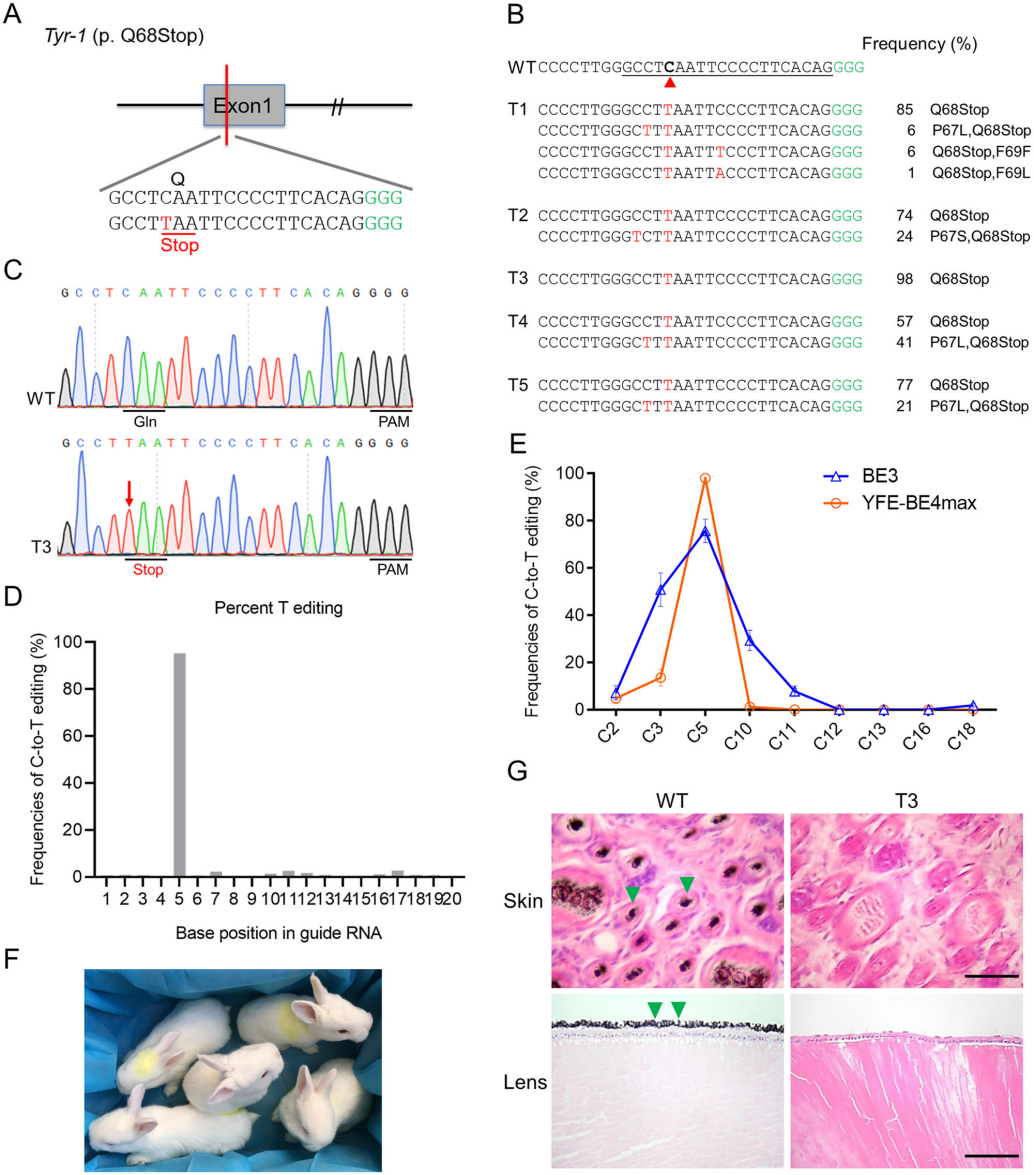
Generation of an albinism rabbit model using the YFE-BE4max system. **a** The target sequence at the *Tyr* p. Q68stop locus. Target sequence (black), PAM region (green). The target C-to-T-editing sites or potential bystander mutations are marked in red or blue, respectively. **b** Alignments of mutant sequences from targeted deep sequencing. Target sequence (underlined), PAM region (green), and substitutions (red). The column on the right indicates the frequencies of mutant alleles. WT, wild type. **c** Representative Sanger sequencing chromatograms from WT and *Tyr* mutant rabbits (T3). The red arrow indicates the mutated nucleotide. The relevant codon identities at the target site are presented under the DNA sequence. **d** The predicted editing bar plot based on Sanger sequencing chromatograms from T3 by EditR. **e** Comparison of the editing frequencies of single C-to-T conversions using BE3 and YFE-BE4max at *Tyr-1* in F0 rabbits. **f** All five mutant rabbits exhibited a systemic albino phenotype. **g** H&E staining of skin and lens from WT and *Tyr* mutant (T3) rabbits. The green triangle highlights the melanin. Scale bars in the skin or lens are 50 μm or 100 μm, respectively.

To further demonstrate the use of YFE-BE4max to generate F0 mutant rabbits, we produced albino and premature aging rabbits by mutate *Tyr* and *Lmna* gene, respectively. The *TYR* gene is the causal gene of human oculocutaneous albinism type 1 (OCA1)^15^, a single C·G to T·A base pair conversion was designed in exon 1 of the rabbit *Tyr* gene for the purpose of yielding a premature stop codon (p. Q68stop) (Fig. 3a). After microinjection of YFE-BE4max-encoding mRNA and single guide RNA (sgRNA), rabbit embryos were transplanted into surrogate mother. Five pups were finally obtained, and the result of targeted deep sequencing showed that all these newborn rabbits (100%) were homozygous with nonsense mutation at the target site (Fig. 3b and Table 1). Notably, the T3 mutant harbored only the mutant allele at target C5 to induce the completely precise p. Q68stop mutation of the rabbit *Tyr* gene (Fig. 3b–d), which is arduous for BE3 to achieve due to rA1’s excessive bystander activity^6^. Though slight bystander mutations (4.8% at C2, 13.6% at C3, and 1.2% at C10) proximal to the targeted C5 were also observed, the frequency of unwanted mutation was significantly reduced by YFE-BE4max in compare with BE3^6^ (Fig. 3e). Moreover, few C-to-A substitutions were also observed in one mutant rabbit (1%, T1), whereas no indels were detected, further demonstrating the high product purity of YFE-BE4max (Fig. 3b). No obvious off-target mutations were detected at potential off-target sites in mutant rabbits by using Sanger sequencing (Fig. S6a). All five pups (100%) showed a systemic albino phenotype (Fig. 3f). In addition, histological haematoxylin and eosin (H&E) staining revealed the absence of melanin in the skin and lens of mutants, but not in the wild type (WT) rabbit (Figs. 3g and S4). These results showed efficient base substitution at *Tyr-1* by the YFE-BE4max and its potential to decrease unwanted bystander mutations in rabbits.

**Table 1:** Generation of targeted mutations in F0 rabbits.

System	Target site	Embryos transferred	No. of offspring	Mutant ratio (%)			
				No. of mutants	Frequencies of precise target editing	No. of indels	No. of non-C-to-T editing
YFE-BE4max	*Tyr-1*	34	5	5 (100)	(57–98)	0 (0)	1 (20)
	*Lmna-1*	42	6	6 (100)	(57–98)	0 (0)	0 (0)

### Base conversion at *Lmna* to precisely mimic the human HGPS mutation

**Fig. 4:**
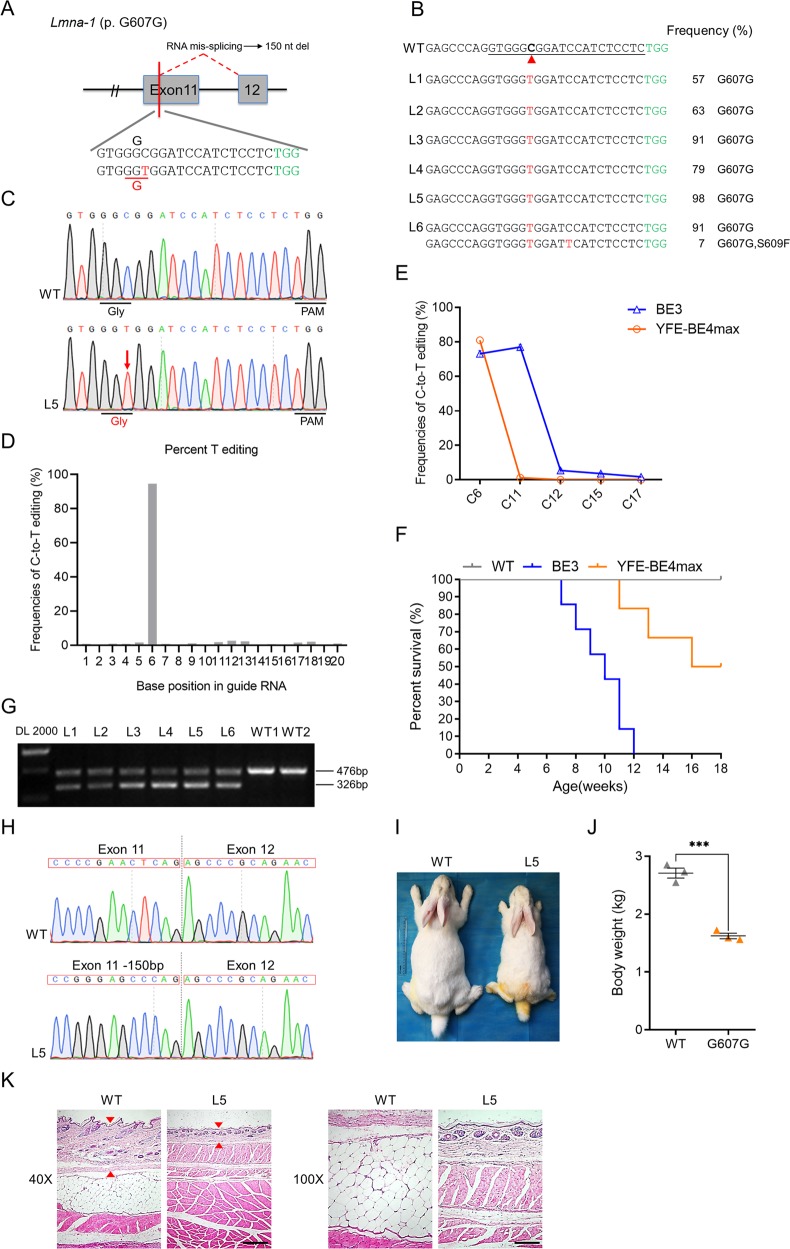
Generation of an HGPS rabbit model using the YFE-BE4max system. **a** The target sequence at the *Lmna* p. G607G locus. Target sequence (black), PAM region (green). The targeted C-to-T-editing site and potential bystander mutations are marked in red or blue, respectively. **b** Alignments of mutant sequences from targeted deep sequencing. Target sequence (underlined), PAM region (green), and substitutions (red). The column on the right indicates the frequencies of mutant alleles. WT, wild type. **c** Representative Sanger sequencing chromatograms from WT and *Lmna* mutant rabbits (L5). The red arrow indicates the mutated nucleotide. The relevant codon identities at the target site are presented under the DNA sequence. **d** The predicted editing bar plot based on Sanger sequencing chromatograms from L5 by EditR. **e** Comparison of the editing frequencies of single C-to-T conversions using BE3 and YFE-BE4max at *Lmna-1* in F0 rabbits. **f** Survival curves of WT and F0 mutant rabbits produced by the BE3 and YFE-BE4max systems. **g** Demonstration of the abnormal splicing product using RT-PCR, showing a spliced product of 326 bp in mutants (L1–L6) rather than WT rabbits. **h** Sanger sequencing chromatograms of the abnormal RT-PCR product confirmed the deletion of 150 nucleotides within exon 11 in the mutant rabbit (L5). **i** Photograph of WT and *Lmna* mutant (L5) rabbits at 3 months. **j** Quantification and comparison of the body weight between WT and *Lmna* mutant rabbits at 3 months. ***p < 0.001, Student’s *t* test. **k** H&E staining of the skin from a 3-month-old *Lmna* mutant (L5) compared with that of a WT rabbit. The red triangle highlights the thin skin. Scale bars at ×40 and ×100 are 500 μm and 200 μm, respectively.

A de novo point mutation (p. G608G) in the *LMNA* gene can lead human classical Hutchinson–Gilford progeria syndrome (HGPS), which is a rare genetic disorder that characterized by premature and rapid aging shortly after birth^16,17^. Here, a single C-to-T conversion in exon 11 of *Lmna* gene was designed to induce p.G607G mutation in rabbit, which is equivalent to the p.G608G mutation in human HGPS^6^ (Fig. 4a). Notably, targeted point mutation at the target C6 was successfully induced all of six (100%) rabbits with editing frequencies from 57 to 98% (Fig. 4b and Table 1). Moreover, a few bystander mutations were only detected in the L6 mutant (7% at C11) (Fig. 4b). In particular, the L5 mutant harbored only the desired C-to-T conversion at target C6 with 98% mutant frequency, enabling it to induce highly efficient and accurate p. G607G mutation (Fig. 4b–d). The analysis of deep sequencing results showed that YFE-BE4max significantly reduced bystander mutations at the *Lmna* locus in compare with previous BE3 system (Fig. 4e). Similarly, no indels, non-C-to-T conversions or off-target mutations were detectable in all mutant rabbits (Figs. 4b and S6b).

Mutations in the *LMNA* gene are associated with a wide range of human genetic disorders, and different mutations may cause discrepant phenotypes^18^. BE3 system is likely to induce high frequency of bystander mutation at C11 thus mediating p. S609F missense mutation, which may potentially affect the phenotype of mutants. Actually, the all F0 mutant rabbits produced by BE3 died within 12 weeks due to a lethal phenotype, but three mutant rabbits generated by YFE-BE4max were still alive at 16 weeks to date (Fig. 4f). This result indicated that the inaccurate p. G607G mutation with high frequencies of bystander mutations using BE3 could not faithfully reflect the phenotype of human HGPS. The p.G607G mutation results in the deletion of 150 nucleotides (50 aa) at the C-terminal of exon 11 by creating a cryptic splice donor site, thus producing a mutant lamin A protein, termed “progerin”^16^. As expect, the RT-PCR analysis and subsequent Sanger sequencing showed the presence of truncated products with 150 nucleotides deletion in mutants, which do not exist in WT rabbits (Fig. 4g, h, and S5). Furthermore, the typical phenotype of marked reductions in growth rate and survival rate (Fig. 4f), short stature (Fig. 4i, j), thin skin, and loss of subcutaneous fat (Fig. 4k) were also observed in the mutants. These observations are completely consistent with RNA mis-splicing and corresponding phenotypes observed in human HGPS patients^16,17^. Over all, with great editing efficiency and minimized bystander activity, YFE-BE4max successfully mediated *Lmna-1* mutation in F0 rabbit, which precisely recapitulate pathological features of classical human HGPS.

## Discussion

In this study, we demonstrated that the YE base editors make a compromise between efficiency and precision, and especially YEE base editors significantly reduce editing efficiency. To overcome this limitation, we developed an optimized rA1-nCas9 fusion, YFE-BE4max, effectively narrow the editing width to as little as ~3 nu while maintaining high efficiency in rabbits by rationally engineering the deaminase domain. In addition, the Y120F-BE4max can efficiently induce C-to-T conversions with narrowed ~3–4 nu windows, similar to YE1-BE4max. Moreover, YFE-BE4max was used to induce site-specific single-base substitutions with 100% efficiency and reduced bystander mutations in generating F0 rabbits at *Tyr-1* p. Q68stop and *Lmna-1* p. G607G loci. These results indicate that these efficient and precise base editors can be used as a reliable tool for inducing high-precision base editing in rabbits. Furthermore, a similar strategy can be widely used in other organisms in the future.

In addition to accuracy, the genome-targeting scope also represents a primary obstacle for base editors. To date, a variety of SpCas9 homologs and variants that recognize a variety of PAMs were found, such as SaCas9 (NNGRRT)^19^, Cpf1 (TTTV)^20^, ScCas9 (NNG)^21^, Nme2Cas9 (N4CC)^22^, SpCas9-VQR (NGA)^23^, and SpCas9-NG (NGN)^24^. It may further improve the accuracy and genome-targeting scope of base editing to combine these variants with the YFE system.

Moreover, a novel BE architecture with an engineered human APOBEC3A (eA3A) domain offered an alternative strategy to further reduce bystander mutations, but the application of this system was restricted by the presence of TCR (A/G) motifs^5^. Context-dependent base editors represent an important advance that offers more precise base editing, whereas may lower the target site applicability because the target nucleotide must naturally exist in the preferred sequence context^25^. Therefore, selecting reasonable base editors with narrowed window or context-specificity to preferentially edit the target base over the bystander base may be the future approach.

In summary, our optimized YFE system is a precise base editing tool that has significantly reduced bystander activity and high editing efficiency. In addition, YFE system could be used to precisely mimic human pathogenic mutations by inducing C-to-T base conversions in rabbits. Overall, these engineered Cas9-cytidine deaminase fusions are promising tools for animal model establishment and precise gene therapy in the future.

## Materials and methods

### Ethics statement

New Zealand white and Lianshan black rabbits were obtained from the Laboratory Animal Center of Jilin University (Changchun, China). All animal studies were conducted according to experimental practices and standards approved by the Animal Welfare and Research Ethics Committee at Jilin University (IACUC number: KT201801001).

### Plasmid construction

The BE4-Gam and BE4max plasmids were obtained from Addgene (#100806 and #112093). The corresponding mutations were introduced into BE4-Gam or BE4max to obtain YE1 base editors (W90Y + R126E in rA1), YEE base editors (W90Y + R126E + R132E in rA1), Y120F-base editors (Y120F in rA1) and YFE-base editors (W90Y + Y120F + R126E in rA1). Plasmid site-directed mutagenesis was performed using the Fast Site-Directed Mutagenesis Kit (TIANGEN, Beijing). All site-directed mutation primers are listed in Table S1.

### mRNA and gRNA preparation

All plasmids were linearized with NotI and transcribed in vitro using the HiScribe T7 ARCA mRNA Kit (NEB). The RNeasy Mini Kit (Qiagen) was used for mRNA purification according to the manufacturer’s instructions. The sgRNA oligos were annealed into pUC57-sgRNA expression vectors containing a T7 promoter. The sgRNAs were then amplified and transcribed in vitro using the MAXIscript T7 Kit (Ambion) and purified using the miRNeasy Mini Kit (Qiagen) according to the manufacturer’s instructions. The sgRNA oligo sequences used in this study are listed in Table S2.

### Microinjection of rabbit zygotes and embryo transfer

The protocol used for the microinjection of pronuclear-stage embryos has been described in detail in our previously published study^26^. In brief, a mixture of mRNA (200 ng/μl) and sgRNA (50 ng/μl) was co-injected into the cytoplasm of pronuclear-stage zygotes. The injected embryos were transferred into Earle's Balanced Salt Solution (EBSS) medium for short-term culture at 38.5 °C, 5% carbon dioxide and 100% humidity. Then, ~30–50 injected zygotes were transferred into the oviducts of recipient rabbits.

### Single-embryo PCR amplification and rabbit genotyping

Each group of base editing was injected with BE-encoding mRNA and corresponding sgRNA using an average of 10 embryos to test the base editing efficiency. The injected embryos were transferred into EBSS medium for culture at 38.5 °C, 5% carbon dioxide, and 100% humidity. Then, the injected embryos were collected at the blastocyst stage. Genomic DNA was extracted in embryo lysis buffer (1% NP40) at 56 °C for 60 min and then at 95 °C for 10 min in a BIO-RAD PCR Amplifier. Then the extracted products were amplified by PCR (95 °C, 5 min, 42 cycles of (95 °C, 30 s, 58 °C, 30 s, 72 °C, 30 s), 72 °C, 5 min) and determined by Sanger sequencing. The Sanger sequencing result of each blastocyst was used to evaluate base editing frequencies or indel frequencies by EditR^8^. The genomic DNA of newborn rabbits was extracted from ear clips and analyzed by PCR genotyping, Sanger sequencing and targeted deep sequencing. All primers used for genotyping are listed in Table S3.

### Targeted deep sequencing

Targeted sites were amplified from genomic DNA using Phusion polymerase (Thermo Fisher Scientific). The paired-end sequencing of PCR amplicons was performed by Sangon Biotech (Shanghai) using an Illumina MiSeq. The average depth of coverage for the sequencing runs was ~20,000 reads.

### Off-target assay

The top ten potential off-target sites in the rabbit genome for sgRNA were predicted to analyze site-specific edits according to Cas-OFFinder^27^ (http://www.rgenome.net/cas-offinder/). All primers for the off-target assay are listed in Tables S4 and S5.

### H&E staining

The dorsal skin and lens from WT and mutant rabbits were fixed in 4% paraformaldehyde for 48 h, embedded in paraffin wax and subsequently sectioned for slides. The slides were stained with H&E and viewed under a Nikon TS100 inverted microscope.

### Statistical analysis

All data are expressed as the mean ± SEM of at least three individual determinations for all experiments. Data were analyzed by Student’s *t* test via GraphPad prism software 6.0. Probability values < 0.05 (*p* < 0.05) were considered to be statistically significant. *p < 0.05, **p < 0.01, ***p < 0.001, ****p < 0.0001.

## Supplementary information

Supplementary materials

## Data Availability

The authors state that all data necessary for confirmation of the conclusions presented in the article are represented fully within the article or are available from the authors upon request.
