# Supplementary material for: Efficient base editing with high precision in rabbits using YFE-BE4max
Source: Cell Death Dis. 2020 Jan 20;11(1):36. doi: 10.1038/s41419-020-2244-3 (PMC6971250; doi:10.1038/s41419-020-2244-3)
Supplement: Supplementary file 1 — Supplementary materials [file 41419_2020_2244_MOESM1_ESM.docx]

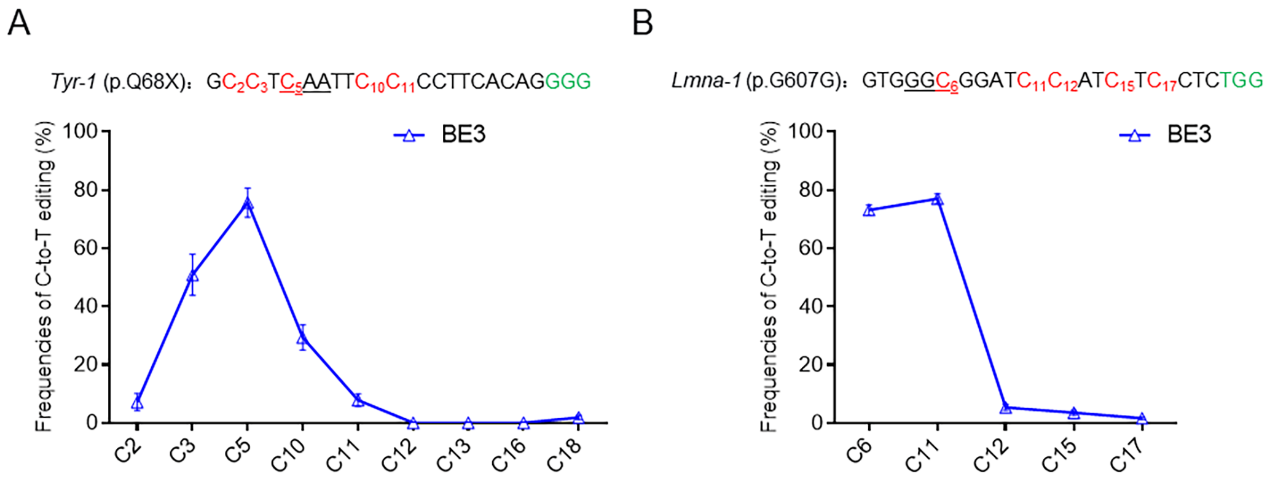


**Figure S1.** Analysis of single C-to-T conversion frequency of BE3 at *Tyr-1* and *Lmna-1* in founder (F0) rabbits. The deep sequencing data used for analysis are sourced from our previous report[^1^](#_ENREF_1). Target sequence (black), PAM region (green), mutated Cs (red, counting the PAM as positions 21-23), target mutant amino acid (underlined).


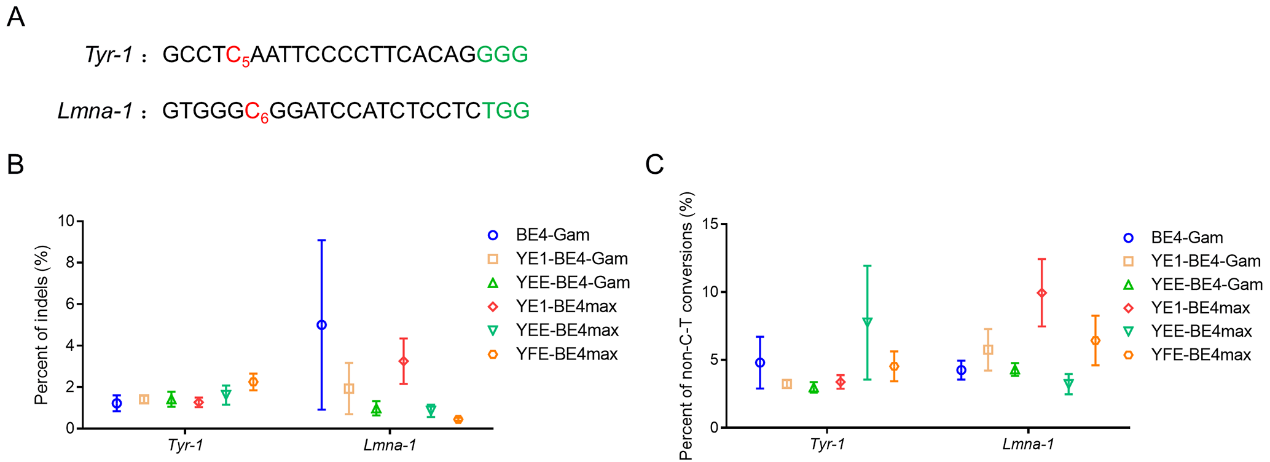


**Figure S2.** By-product formation in base editing mediated by six base editors. (**A**) The target sequence at *Tyr-1* and *Lmna-1*. PAM region (green), target C (red, counting the PAM as positions 21–23). (**B**, **C**) Frequencies of indels and non-C-to-T conversions at *Tyr-1* and *Lmna-1* by six base editors in rabbit embryos.


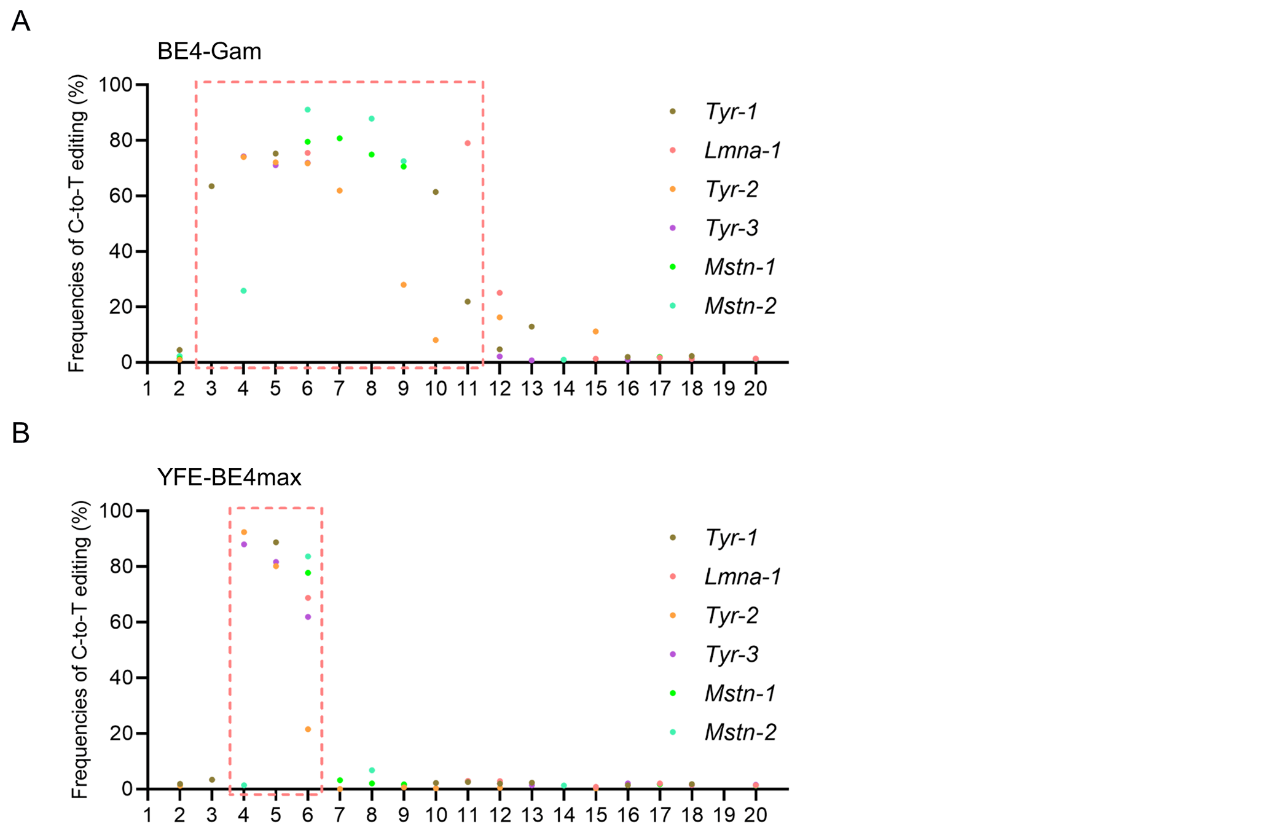


**Figure S3.** Summary of the base editing frequency at each cytosine in the spacer region for the indicated six sgRNAs using BE4-Gam (**A**) and YFE-BE4max (**B**). These data show that the major editing window ranges of BE4-Gam and YFE-BE4max from the position 3 to 11 or from the position 4 to 6 in spacer region, respectively.


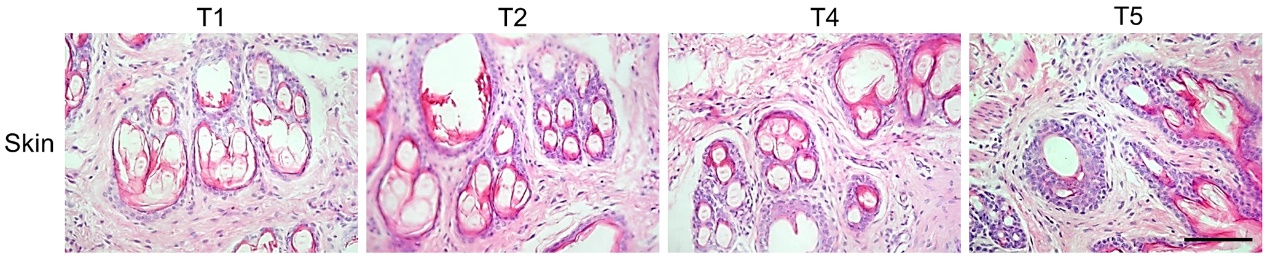


**Figure S4.** H&E staining of skin from *Tyr* mutant (T1, T2, T4 and T5) rabbits at 3 months. No obvious melanin was observed in the skin of all detected mutants. Scale bars are 100 μm.

**
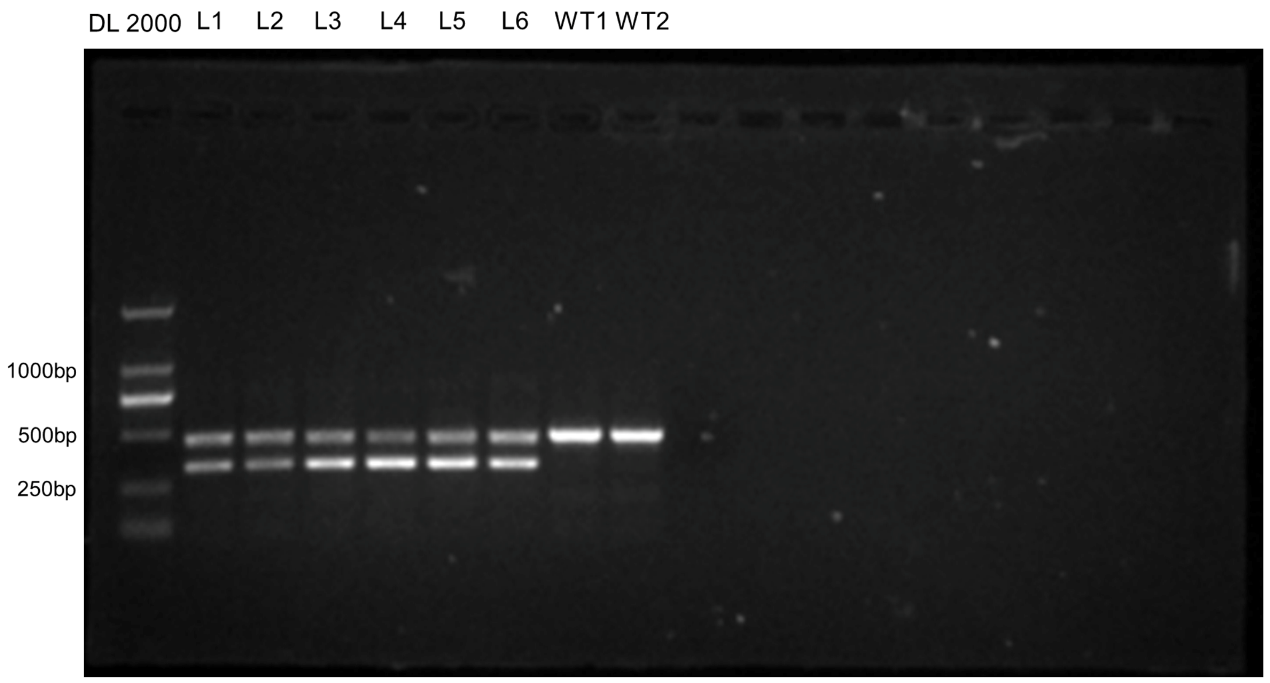
**

**Figure S5.** Uncropped gels corresponding to Fig. 4G. Abnormal splice products were demonstrated by RT-PCR, showing that mutant rabbits possess 326bp spliced products due to the activation of the cryptic splice sites.


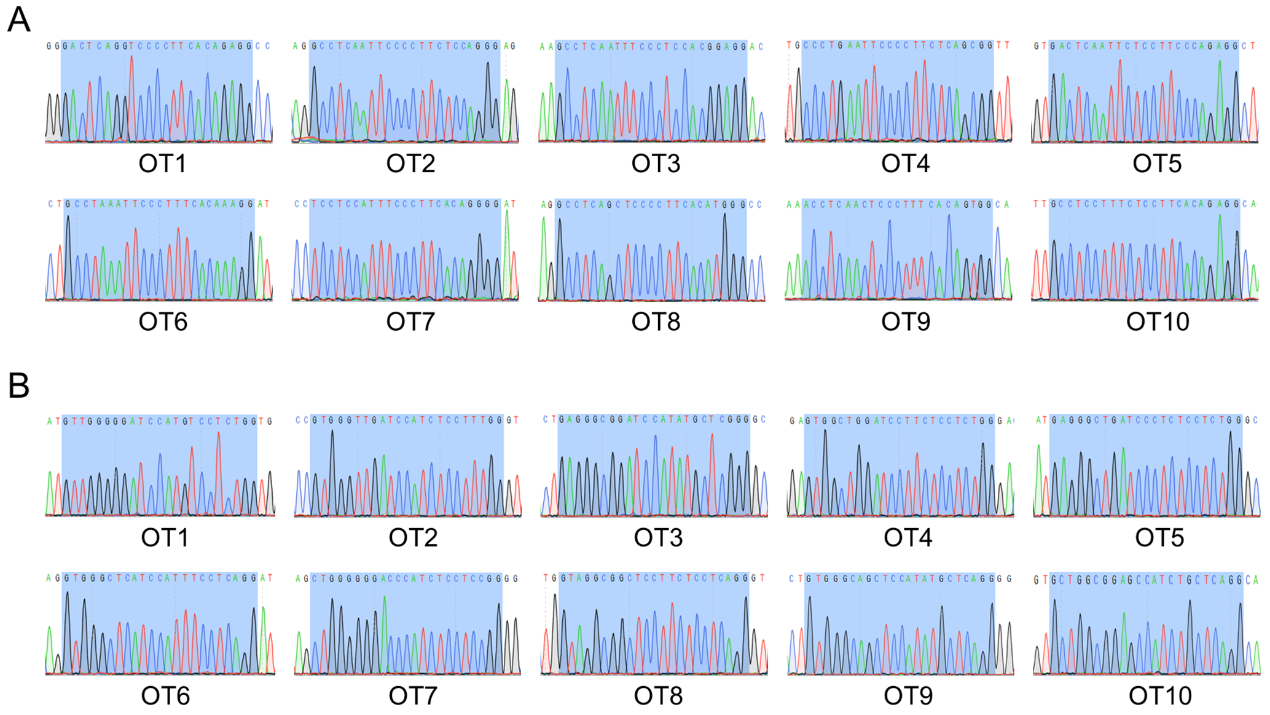


**Figure S6.** Off-target detection in F0 mutant rabbits. Chromatogram sequence analysis of POTs using PCR products for the *Tyr-1*(**A**) and *Lmna-1* (**B**). A total of 20 bp of the POTs and the PAMs are highlighted.

**Table S1.** Primers used for site-directed mutation in this study.

| **Plasmid template** | **Mutation** | **Primers (5’-3’)** |
| --- | --- | --- |
| BE4-Gam | W90Y (rA1) | F: ACCTGGTTTCTCAGCTATAGCCCATGCGGC  R: ATAGCTGAGAAACCAGGTAATGCTGCACCT |
|  | R126E (rA1) | F: CACCACGCTGACCCCGAGAATCGACAAGGC  R: CTCGGGGTCAGCGTGGTGGTACAGCCTTGC |
|  | R132E (rA1) | F: AATCGACAAGGCCTGGAAGATTTGATCTCT  R: TTCCAGGCCTTGTCGATTCTCGGGGTCAGC |
|  | Y120F (rA1) | F: TACATCGCAAGGCTGTTCCACCACGCTGAC  R: AACAGCCTTGCGATGTAAATAAACAGAGTG |
| BE4max | W90Y (rA1) | F: TGGTTTCTGAGCTACTCCCCTTGCGGAGAG  R: GTAGCTCAGAAACCATGTGATGCTACATCT |
|  | R126E (rA1) | F: CACCACGCAGACCCAGAGAATAGGCAGGGC  R: TCTGGGTCTGCGTGGTGATACAGCCTGGCG |
|  | R132E (rA1) | F: AATAGGCAGGGCCTGGAGGATCTGATCAGC  R: CTCCAGGCCCTGCCTATTCTCTGGGTCTGC |
|  | Y120F (rA1) | F: ACATCGCCAGGCTGTTTCACCACGCAGACC  R: AACAGCCTGGCGATGTAGATAAACAGTGTC |

**Table S2.** The two oligonucleotide strands used to construct the pUC57-sgRNA vectors.

| **Target site** | **Oligonucleotide 1** | **Oligonucleotide 2** |
| --- | --- | --- |
| *Tyr-1* | TAGGCCTCAATTCCCCTTCACAG | AAACCTGTGAAGGGGAATTGAGG |
| *Lmna-1* | TAGGTGGGCGGATCCATCTCCTC | AAACGAGGAGATGGATCCGCCCA |
| *Tyr-2* | TAGGCTCCCCTCCTCATCAGATG | AAACCATCTGATGAGGAGGGGAG |
| *Tyr-3* | TAGGAGTCCCTGTGGCCAGCTCTC | AAACGAGAGCTGGCCACAGGGACT |
| *Mstn-1* | TAGGCAAACCCCAGAGGTTCAGC | AAACGCTGAACCTCTGGGGTTTG |
| *Mstn-2* | TAGGACACTCTCCAGAGCAGTAAT | AAACATTACTGCTCTGGAGAGTGT |

**Table S3.** Primers used for genotyping in this study.

| **Target site** | **Primers** | **Sequence (5’-3’)** | **Product size (bp)** |
| --- | --- | --- | --- |
| *Tyr-1* | *Tyr-1*-F  *Tyr-1*-R | ATCCGCTCAAGCAGGTATTG GACATAGTCTGGGCTCGTAGTA | 487 |
| *Lmna-1* | *Lmna-1*-F  *Lmna-1*-R | AGCACCCAAGCCACAAAT  CACAAAGGCATGACTCACCT | 490 |
| *Tyr-2* | *Tyr-2*-F  *Tyr-2*-R | GTGGTGGATGCAAGACTAGAA  AGCTGAAATTGGCAGCTTTG | 408 |
| *Tyr-3* | *Tyr-3*-F  *Tyr-3*-R | ATCCGCTCAAGCAGGTATTG GACATAGTCTGGGCTCGTAGTA | 487 |
| *Mstn-1* | *Mstn-1*-F  *Mstn-1*-R | TAGAGGTCAAGGTAACGGACA  GAGACATCTTTGTGGGAGTACAG | 282 |
| *Mstn-2* | *Mstn-2*-F  *Mstn-2*-R | TAGAGGTCAAGGTAACGGACA  GAGACATCTTTGTGGGAGTACAG | 282 |

**Table S4.** The primers used for identifying potential off-target sites of *Tyr-1*. The

mismatched nucleotides are shown in red.

| **Potential Off Target Site** | **Number of mismatches** | **Position** | **PCR Primer** |
| --- | --- | --- | --- |
| GACTCAGGTCCCCTTCACAG  AGG | 3 | chr13:-134185543 | OT1-F: TTCTCTCCTCTCCGCATTCT  OT1-R: TGATGTCAGAGGCCGTCTAT |
| GCCTCAATTCCCCTTCTCCA  GGG | 3 | chr14:-32031659 | OT2-F: CCACAAGCCCTGTAGAATCA  OT2-R: GGAACGCTTATGCCCATTTC |
| GCCTCAATTTCCCTCCACGG  AGG | 3 | chr16:-44146399 | OT3-F: GCAATAGCTTTGTGGCCTTG  OT3-R: ACCCATGTGAGATGCTGATG |
| CCCTGAATTCCCCTTCTCAG  CGG | 3 | chr2:+112817702 | OT4-F: CAGAGGAGACAAAGCAACAATTC  OT4-R: CCCGTCACCATCCCATTAC |
| GACTCAATTCTCCTTCCCAG  AGG | 3 | chr3:+55437364 | OT5-F: CAGGCTCAAAGGTGGTATGA  OT5-R: GAGGACTCTGTGCTTGTTAGAG |
| GCCTAAATTCCCTTTCACAA  AGG | 3 | chr5:-17790033 | OT6-F: TGCCTGTGGGTAATGGTTAG  OT6-R: CCCTAGTCTGAGTGCCAATTTA |
| TCCTCCATTTCCCTTCACAG  GGG | 3 | chr7:-99342691 | OT7-F: CACTCGTGGAGTTTCCTGAAT  OT7-R: AGAAGAAGAAGAGGAGGAGGAG |
| GCCTCAGCTCCCCTTCACAT  GGG | 3 | chr9:-21114580 | OT8-F: TGCCATCACCTAAAGGCTAAG  OT8-R: TGTGGGTCTGTAAGCTCTTTG |
| ACCTCAACTCCCTTTCACAG  TGG | 3 | chr9:-29521405 | OT9-F: AGCTGGGTGATAGGGATACA  OT9-R: CCAACCGAGCCTGGATATTT |
| GCCTCCTTTCTCCTTCACAG  AGG | 3 | chrX:-46475662 | OT10-F: TCTGAAAGGGAGTCAAGGAAAC  OT10-R: TCTGCCAACAGCCTGAAAT |

**Table S5.** The primers used for identifying potential off-target sites of *Lmna-1*. The

mismatched nucleotides are shown in red.

| **Potential Off Target Site** | **Number of mismatches** | **Position** | **PCR Primer** |
| --- | --- | --- | --- |
| GTTGGGGGATCCATGTCCTCTGG | 3 | chr16:-57593577 | OT1-F: TGGAGGTTTCCAGGTGATTTC  OT1-R: CCATCAGTGCCAGTCAGATAAA |
| GTGGGTTGATCCATCTCCTTTGG | 3 | chr17:+74437313 | OT2-F: ACAGCACGTTCGGTGTATT  OT2-R: GTCCTCTCCTGAAATCGCTAAA |
| GAGGGCGGATCCATATGCTCGGG | 3 | chr16:+33491075 | OT3-F: GTGAATGTGGGAGCCTTGTT  OT3-R: GGTAGGTTTGTTCAGGTCCATTAG |
| GTGGCTGGATCCTTCTCCTCTGG | 3 | chr3:-15323034 | OT4-F: AAGCTATCCCTGCTCCTGTA  OT4-R: AGACCCAACTCTACCCAAGA |
| GAGGGCTGATCCCTCTCCTCTGG | 3 | chr5:-33291858 | OT5-F: CAGGCACTGTGTCAACTACTTA  OT5-R: ATGGATGATGGCAGAAAGACA |
| GTGGGCTCATCCATTTCCTCAGG | 3 | chrUn0027:  -1219872 | OT6-F: CAGTCTCCTGGTCTCTGTAGT  OT6-R: TATGTTTGAGGCGTTCCTGAG |
| CTGGGGGGACCCATCTCCTCCGG | 3 | chrUn0148:  +586207 | OT7-F: CCGCCTGTACCAACAATCA  OT7-R: GTACCGCTGTGCAGAGAAA |
| GTAGGCGGCTCCTTCTCCTCAGG | 3 | chrUn0869:  -31704 | OT8-F: TTCAGCGGAGGTCGTTTG  OT8-R: TTGCTAAAGGTTCCAGAGAGAC |
| GTGGGCAGCTCCATATGCTCAGG | 4 | chr1:-51997899 | OT9-F: CTCTTGAGTCTGGCTGAGATAAC  OT9-R: CTATTCAGTGCTCCCGTGTTAG |
| GCTGGCGGAGCCATCTGCTCAGG | 4 | chr1:-79219953 | OT10-F: TGTGACAGGTACGGCAAATTA  OT10-R: GACATCCTGACCAGAGGAAAC |

**References**

1. Liu, Z. et al. Highly efficient RNA-guided base editing in rabbit. *Nature communications* **9**, 2717 (2018).
